# Supplementary material for: Goal commitment is supported by vmPFC through selective attention
Source: Nat Hum Behav. 2024 Apr 17;8(7):1351–65. doi: 10.1038/s41562-024-01844-5 (PMC11272579; doi:10.1038/s41562-024-01844-5)
Supplement: Supplementary file 1 — Supplementary Methods. [file 41562_2024_1844_MOESM1_ESM.pdf]

nature human behaviour

Article

https://doi.org/10.1038/s41562-024-01844-5

Goal commitment is supported by vmPFC through selective attention

In the format provided by the authors and unedited

Model validation process

To validate the model selection procedure, we performed a model recovery analysis to confirm that the competing models were distinguishable within the empirical parameter range.[1] In addition to fitting the four basic normative models described above (see 'Fitting Normative Model'), we also tested the recoverability of the basic models plus goal progress (See 'Goal Progress' section in Methods), which we found to have an additional impact on behaviour:

1. $SV_{abandon} = \beta_0 + \beta_1 * (alternative\ value_{offer-max} - goal\ value_{offer-max}) + \beta_3 * goal\ progress$

2. $SV_{abandon} = \beta_0 + \beta_1 * (alternative\ value_{myopic} - goal\ value_{myopic}) + \beta_3 * goal\ progress$

3. $SV_{abandon} = \beta_0 + \beta_1 * (alternative\ value_{prospective} - goal\ value_{prospective}) + \beta_3 * goal\ progress$

4. $SV_{abandon} = \beta_0 + \beta_1 * (alternative\ value_{tree-search} - goal\ value_{tree-search}) + \beta_3 * goal\ progress$

We used the empirical parameters from logistic regression models which were fit separately to each participant to simulate choices for each model. A soft-max function was then used to simulate choices from the subjective value:

$$P_{abandon} = \frac{1}{1 + e^{-\mathrm{SV}_{abandon}}}$$

Subsequently, all models were fitted to all simulated datasets, and model BICs used to select the best fitting model. To account for stochasticity resulting from the soft-max function, we repeated the simulation process 100 times for each of the 30 participants (resulting in 3000 simulated datasets per model). The averaged confusion matrix is displayed in Extended Data fig.3c, showing that each simulated model can be correctly identified during model recovery. Importantly, we find that in the empirical parameter range and across 100 repetitions, there are no cases of more simple models being confused for the empirically best-fitting model (tree-search model).

Persistence bias parameter recovery and test-retest reliability

We investigated the test-retest reliability of the persistence bias parameters across the two testing sessions (inside and outside the scanner), by using the intraclass correlation (ICC). The ICC captures the agreement across measurements while allowing for baseline differences across sessions.[2] We used the ICC(2k) score (absolute-agreement, two-way random-effects model) as used in similar paradigms[3] where the conventional approach considers scores below 0.5 as 'low', between 0.5 and 0.75 as 'moderate', and above 0.75 as 'good'.[4] ICC estimates were calculated using Pingouin statistical package[5] (version 0.5.3.) For all future analyses involving persistence biases, we used the values fit to the aggregated data across

both sessions unless explicitly indicated otherwise. Persistence biases across the two sessions are shown in Extended Data fig.4d.

We also investigated the reliability of the two sub-parameters from which persistence bias is derived (intercept and slope) using both simulated parameter recoveries and test-retest correlation across the two behavioural testing sessions (inside and outside the scanner). All three parameters show robust recovery in simulated data, as well as significant test-retest reliability in empirical data across the two behavioural sessions, as shown in Extended Data fig.4 (note that persistence biases have higher recoverability and test-retest reliability than either subcomponents on their own).

Impact of goal progress on persistence

We define goal progress as the proportion of the current goal completed (i.e. current net contents / net size; Extended Data fig.3b). To quantify the additional impact of goal progress on peoples' choices, we used chi-squared tests to determine whether each additional regressor improved our basic mixed-effects model across participants. For each model, the intercept and slopes for every regressor in the models were also included as random effects across participants.

5. $$SV_{abandon} = \beta_0 + \beta_1 * V_{abandon}$$

6. $$SV_{abandon} = \beta_0 + \beta_1 * V_{abandon} + \beta_2 * goal\ progress$$

7. $$SV_{abandon} = \beta_0 + \beta_1 * V_{abandon} + \beta_2 * goal\ progress + \beta_2 * goal\ progress * V_{abandon}$$

After finding that value is used less over goal progress (model 3 above), we asked how this related to our attentional hypothesis: specifically, that people will ignore alternative goal value more than current goal value as attention is increasingly oriented towards the current goal. We split the aggregate value of abandonment into its two components associated with the current and best alternative goals. We fit this final model capturing asymmetry in value use at the level of individual participant. For each participant, we fit a logistic regression model which included the interaction between each source of value and goal progress:

8. $$SV_{abandon} = \beta_0 + \beta_1 * goal\ value_{tree-search} + \beta_2 * alternative\ value_{tree-search} + \beta_3 * goal\ progress + \beta_4 * goal\ progress * goal\ value_{tree-search} + \beta_5 * goal\ progress * alternative\ value_{tree-search}$$

Since the beta coefficient distributions did not violate tests of normality, t-tests were used to determine whether there was a significant difference between the disappearance of current-goal and alternative-goal value across goal pursuit, using the coefficients found for each individual. We first tested whether the interaction between value and goal progress was different from zero separately for current goal and alternative goal value, indicating a change in uses of both value components across goal pursuit. We then tested for the difference in slopes between these coefficients (difference in betas for best-alt*goal-progress and for sign-flipped current-goal*goal-progress). Note we use the *sign-flipped* coefficients for current-goal*goal-progress because the value of the current goal and the value of the best alternative have opposing impact on the likelihood of switching (see Fig.2d). See Extended Data Fig.5c

for an illustration of this analysis in simulated normative behaviour, showing this effect is not an artefact of the experimental design.

Region of interest analyses

ROI extraction procedure

ROI extraction involved selecting peaks either for activity related to the contrast capturing the value of persisting (current goal value–best alternative value; peaks in vmPFC and ventral striatum), or capturing the value of abandonment (best alternative value–current goal value; dACC), following cluster correction (Illustration of ROIs in Extended Data fig.7a,b,c; all activity peaks listed in Extended Data tab.1). Since our whole-brain analysis did not reveal any activation for the value of the third alternative in these areas, we did not include the third alternative in subsequent analyses. Regions of interest consisted of spheres with a 3 voxel radius (7.2mm^3). In time-course analyses, activity in these spheres was up-sampled by a factor of 10, and cut into epochs which were aligned to the onset of the decision phase (see plots of activity time-courses in Extended Data fig.7d,e,f).

Activity in these value-related ROIs was then used to investigate a) the modulation of value signals over the course of goal progress and b) correlations with individual differences in persistence biases. Any time courses displaying non-orthogonal contrasts are for illustration purposes only and no statistical tests were performed.

Baseline activity analysis

Our previous whole-brain analysis found that activity relating to goal progress was present in the inter-trial interval, with the peak of this activity located in vmPFC. Previous research has shown that baseline representations of long-term task variables influence subsequent choice behaviour through vmPFC.[6,7,8] We therefore asked whether this baseline goal-related activity at the onset of decisions was relevant for the behavioural differences in choices and attention we observed.

We quantified individuals' baseline representation of goal progress in the vmPFC ROI (see ROI selection and extraction procedure). As in previous paradigms[7], we define baseline activity as the activity present at the onset of the choice offers, before the new offers or decision itself influence the dynamics (i.e. t=0 of the time course shown in Extended Data fig.7d,e,f). We predicted vmPFC baseline activity in a model with all the identical regressors to those listed in the whole brain analysis (see *Decision Time analysis).* Then we specifically tested for a relationship between the beta-weight for goal progress (proportion of goal completed) and our behavioural measures (persistence bias and goal-directed attention). Spearman's correlation was used because both the neural activity betas and persistence bias distributions violated the assumption of normality.

To test the specificity of our vmPFC baseline effect we did three additional control analyses. First, we showed baseline representations of goal progress in the other two ROIs (ventral striatum and ACC) do not predict persistence biases (Extended Data fig.8b). Second, we tested whether the effect was specifically driven by baseline rather than decision-related activity. We showed persistence biases were not predicted by goal-related activity time-locked to the decision itself (Extended Data fig.8c). In addition, we show the relationship

between baseline vmPFC and behaviour is unaffected if baseline activity is extended two seconds prior to choice-onset, supporting the claim that pre-decision activity is the critical predictor of these individual behavioural differences (Extended Data fig.8d).

Value modulation analyses

We found an asymmetry in the use of value in behaviour, where the influence of value related to alternative goals disappeared more than the influence of value related to the current goal, over the course of goal pursuit. Therefore, we asked whether neural representations of value also changed over the course of goal pursuit.

As for behaviour, we predicted neural activity using the interaction between goal progress and each source of value (tree-search model value of best alternative and tree-search model value of current goal). We included regressors for the main effects as well as additional regressors controlling for switch choices and response times (log RT). All regressors were normalised before fitting the GLM. To test for statistical significance, we multiplied the fitted beta coefficients for the interaction term (goal progress*value) at each time-point by the double gamma HRF function (also used in the whole brain analysis) and summed the products to produce a coefficient for each participant. We then tested whether there was a significant effect by doing a one-sampled test against 0 (one-sided, for the direction indicating a decreasing impact of value over goal-progress). Non-parametric methods (Wilcoxon signed-rank test) were used because the distribution of beta weights violated the assumption of normality. Note that although we used one-sided tests due to our behavioural prediction of finding a *decreasing* impact of value over goal pursuit, our findings remain the same with two-sided tests.

Spatial task in lesion patients

Our patient group also performed the interleaved spatial task. We quantified spatial attention bias as the accuracy advantage for the current goal item over the alternative item, as described in *Spatial task analyses.* We predicted the vmPFC group would show a lower accuracy advantage for the goal item over the alternative items in the interleaved task, since attention would not be captured by the goal.

While as predicted, the vmPFC group did not show a significant accuracy or reaction time advantage for stimuli related to the current goal (goal item accuracy advantage: mean=0.026, std=0.031, Wilcoxon test for difference against zero: n=5, T=2.0, p=0.188; goal item reaction time advantage: mean=-0.017 , std=0.116, Wilcoxon test for difference against zero: n=5, T=5.0, p=0.625), we cannot interpret this result since we also did not detect goal-oriented spatial attention effects among patients with lesions elsewhere either (goal item accuracy advantage: mean=0.031, std=0.130, Wilcoxon test for difference against zero: n=18, T=80.0, p=0.832; goal item reaction time advantage: mean=0.041 , std=0.104, Wilcoxon test for difference against zero: n=18, T=5.0, p=0.054). Since we were unable to detect goal-oriented attentional biases in either group, there was also no difference in attentional biases between groups (permutation test for difference in goal item accuracy advantage across groups: mean difference=0.004, p=0.464, *n.s.*; permutation test for difference in goal item reaction time advantage across groups: mean difference=0.058, p=0.297, *n.s.*).

A likely explanation for the difficulty detecting attentional biases in the patient cohort compared to the MRI cohort is simply that the fast-paced spatial attention task was too difficult for the older brain-damaged population. In general, this is reflected in accuracy: accuracy among the patient group was considerably worse compared to our MRI participants (mean error in fMRI sample: n=30, mean=0.210 screen units, std=0.026, mean error in patient sample: n=23, mean=0.316, std=0.182; permutation test for difference in means: mean difference=0.106, p=0.002; see Extended Data fig.9e for raw error in each group). In addition, unlike with the MRI cohort, this task was performed remotely with the patients, with likely variation in computer and mouse set-up and internet speed hampering the ability to detect subtle differences in responses in the spatial task. Given we could not detect goal-oriented attentional effects in the lesion patient population for the reasons discussed, we cannot determine whether lesion location affects spatial attention in this study.

Supplementary References

1. Palminteri, S., Wyart, V., & Koechlin, E. (2017). The Importance of Falsification in Computational Cognitive Modeling. *Trends in Cognitive Sciences*, *21*(6), 425–433. https://doi.org/10.1016/j.tics.2017.03.011
2. Shrout, P. E., & Fleiss, J. L. (1979). Intraclass correlations: Uses in assessing rater reliability. *Psychological Bulletin*, *86*(2), 420–428. https://doi.org/10.1037//0033-2909.86.2.420
3. Loosen, A. M., Seow, T., & Hauser, T. U. (2022). *Consistency within change: Evaluating the psychometric properties of a widely-used predictive-inference task*. PsyArXiv. https://doi.org/10.31234/osf.io/qkf7j
4. Koo, T. K., & Li, M. Y. (2016). A Guideline of Selecting and Reporting Intraclass Correlation Coefficients for Reliability Research. *Journal of Chiropractic Medicine*, *15*(2), 155–163. https://doi.org/10.1016/j.jcm.2016.02.012
5. Vallat, R. (2018). Pingouin: statistics in Python. *Journal of Open Source Software*, 3(31), 1026, https://doi.org/10.21105/joss.01026
6. Lopez-Persem, A., Domenech, P., & Pessiglione, M. (2016). How prior preferences determine decision-making frames and biases in the human brain. *ELife*, *5*, e20317. https://doi.org/10.7554/eLife.20317
7. Vinckier, F., Rigoux, L., Oudiette, D., & Pessiglione, M. (2018). Neuro-computational account of how mood fluctuations arise and affect decision making. *Nature Communications*, *9*(1), Article 1. https://doi.org/10.1038/s41467-018-03774-z
8. Abitbol, R., Lebreton, M., Hollard, G., Richmond, B. J., Bouret, S., & Pessiglione, M. (2015). Neural Mechanisms Underlying Contextual Dependency of Subjective Values: Converging Evidence from Monkeys and Humans. *Journal of Neuroscience*, *35*(5), 2308–2320. https://doi.org/10.1523/JNEUROSCI.1878-14.2015
