# Supplementary material for: Goal commitment is supported by vmPFC through selective attention
Source: Nat Hum Behav. 2024 Apr 17;8(7):1351–65. doi: 10.1038/s41562-024-01844-5 (PMC11272579; doi:10.1038/s41562-024-01844-5)
Supplement: Supplementary file 2 — Reporting Summary [file 41562_2024_1844_MOESM2_ESM.pdf]

nature portfolio

Corresponding author(s): Eleanor Holton

Last updated by author(s): 22/01/2024

Reporting Summary

Nature Portfolio wishes to improve the reproducibility of the work that we publish. This form provides structure for consistency and transparency in reporting. For further information on Nature Portfolio policies, see our Editorial Policies and the Editorial Policy Checklist.

Statistics

For all statistical analyses, confirm that the following items are present in the figure legend, table legend, main text, or Methods section.

n/a	Confirmed	
☐	☒	The exact sample size (*n*) for each experimental group/condition, given as a discrete number and unit of measurement
☐	☒	A statement on whether measurements were taken from distinct samples or whether the same sample was measured repeatedly
☐	☒	The statistical test(s) used AND whether they are one- or two-sided<br>*Only common tests should be described solely by name; describe more complex techniques in the Methods section.*
☐	☒	A description of all covariates tested
☐	☒	A description of any assumptions or corrections, such as tests of normality and adjustment for multiple comparisons
☐	☒	A full description of the statistical parameters including central tendency (e.g. means) or other basic estimates (e.g. regression coefficient) AND variation (e.g. standard deviation) or associated estimates of uncertainty (e.g. confidence intervals)
☐	☒	For null hypothesis testing, the test statistic (e.g. *F*, *t*, *r*) with confidence intervals, effect sizes, degrees of freedom and *P* value noted<br>*Give P values as exact values whenever suitable.*
☒	☐	For Bayesian analysis, information on the choice of priors and Markov chain Monte Carlo settings
☒	☐	For hierarchical and complex designs, identification of the appropriate level for tests and full reporting of outcomes
☐	☒	Estimates of effect sizes (e.g. Cohen's *d*, Pearson's *r*), indicating how they were calculated

Our web collection on statistics for biologists contains articles on many of the points above.

Software and code

Policy information about availability of computer code

Data collection	The experimental task used for data collection was custom developed by the authors using PsychoPy Standalone (Version v2021.1.2).
Data analysis	fMRI analyses were carried out using FSL, the FMRIB Software Library (FSL version 6.0.4). Behavioural and further neural analyses were carried out by custom-written scripts in python (3.11.5) and MATLAB (version R2021_a). Code for data analysis can be found in OSF at https://osf.io/mvquk/ (DOI Identifier: DOI 10.17605/OSF.IO/MVQUK.). The following python packages were used for data processing, analysis and visualization: pandas (2.1.1), numpy (1.26.0), seaborn (0.12.2), matplotlib (3.8.0), scipy (1.11.2), statsmodels (0.14.0), pingouin (0.5.3), rpy2 (3.5.11), nilearn (0.10.2), nibabel(5.1.0).

For manuscripts utilizing custom algorithms or software that are central to the research but not yet described in published literature, software must be made available to editors and reviewers. We strongly encourage code deposition in a community repository (e.g. GitHub). See the Nature Portfolio guidelines for submitting code & software for further information.

Data

Policy information about availability of data

All manuscripts must include a data availability statement. This statement should provide the following information, where applicable:

- Accession codes, unique identifiers, or web links for publicly available datasets
- A description of any restrictions on data availability
- For clinical datasets or third party data, please ensure that the statement adheres to our policy

The behavioral data and preprocessed fMRI data has been deposited at OSF (https://osf.io/mvquk/) and is publicly available as of the publication date (DOI Identifier: DOI 10.17605/OSF.IO/MVQUK). The patient lesion maps are not publicly available as this would compromise the privacy of the research participants.

Research involving human participants, their data, or biological material

Policy information about studies with human participants or human data. See also policy information about sex, gender (identity/presentation), and sexual orientation and race, ethnicity and racism.

Reporting on sex and gender	We asked for participants' self-reported gender at the time of data collection: Out of a total of 30 fMRI participants, 19 self-reported as female. We do not include further analysis of gender, as it was not applicable to our research questions.
Reporting on race, ethnicity, or other socially relevant groupings	We did not collect data on race or ethnicity, as it was not applicable to our research questions.
Population characteristics	fMRI participants were majority University students (mean age of 25 years). Lesion patient participants were patients who had previously visited John Radcliffe Hospital Oxford and consented to being contacted (mean age of 58 years). Age-matched control participants were collected from an online recruitment platform, and lived in the UK (mean age of 59 years). All participants had normal or corrected-to-normal vision.
Recruitment	fMRI participants were recruited via email circulation on Oxford University mailing lists and Oxford-based social media platforms. Lesion patients were recruited by email after previously consenting to being contacted for research studies. Age-matched control participants were recruited online via the platform Prolific. There was no self-selection bias.
Ethics oversight	Ethical approval for the fMRI study was obtained by the Oxford Central University Research Ethics Committee (REC; Ref: R72921/RE001).<br>Ethical approval for the patient study was obtained by the London Fullham Research Ethics Committee (IRAS project number: 242551 REC Reference number: 18/LO/2152).

Note that full information on the approval of the study protocol must also be provided in the manuscript.

Field-specific reporting

Please select the one below that is the best fit for your research. If you are not sure, read the appropriate sections before making your selection.

☐ Life sciences ☒ Behavioural & social sciences ☐ Ecological, evolutionary & environmental sciences

For a reference copy of the document with all sections, see nature.com/documents/nr-reporting-summary-flat.pdf

Behavioural & social sciences study design

All studies must disclose on these points even when the disclosure is negative.

Study description	Data are quantitative experimental data. The fMRI study involves imaging data from a 50 minute fMRI scan, and behavioural data during the fMRI scan and in a 30 minute post-scan session. The lesion patient study includes online behavioural data from 23 lesion patients and 27 age-matched healthy controls using the same task paradigm as in the fMRI study.
Research sample	The research sample includes 30 healthy individuals (majority Oxford-based students) for the fMRI study, with mean age=25. This sample is representative of a healthy young population in the UK. For the lesion patient study, the sample included 23 brain-lesioned patients (mean age=58) and 27 age-matched control subjects (mean age=59). These samples are representative of a population of brain-lesioned patients and a population of healthy older individuals in the UK. Importantly, we do not compare behaviour between the younger fMRI group and the older lesion patient group since these samples are not matched. Instead we only compare lesion patient behaviour to the age-matched control population.
Sampling strategy	fMRI sample size (n=30) was chosen as the upper end of the recommended sample size for decision-making imaging studies, based in similar studies of naturalistic decision-making in fMRI paradigms (Juechems et al. 2019, Trudel et al. 2021, Park et al. 2021). Convenience sampling was used.<br>Lesion sample size (n=23) was limited due to patient availability, but is larger than those reported in similar lesion patient studies (Hare et al. 2011, Wolf et al. 2014, Noonan et al. 2017). This sample size is sufficient for recovering the effects we report, based on

	both previous analysis of healthy individuals, and the permutation based size-correction reported in the manuscript. Convenience sampling was used.
Data collection	fMRI study: training and post-scan tasks were undertaken on a laptop in a behavioural testing room, with one experimenter present. fMRI data were acquired at the Oxford Centre for Human Brain Activity using a 3T Siemens scanner. A trained radiographer was present in addition to the experimenter during the fMRI scanning.<br>Lesion patient study: both the lesion sample and the control sample performed the task virtually on a computer at their own home. For the lesion patients, the experimenter was remotely present during the study on the telephone.<br>In both cases, the researcher was not blind to the study hypothesis during data collection, but all training was standardised using computer-based task instructions and computer-based practice questions which were kept consistent across individual sessions.
Timing	Data collection for the fMRI study took place from May 2021 to January 2022 (with data collection limited by shortages in scanner availability at the end of the pandemic).<br>Data collection for the patient study was limited by lesion patient availability, with the first patient tested in May 2021 and the last patient tested in August 2022.
Data exclusions	One fMRI participant was excluded because they withdraw their participation in the study (before taking part in the scan itself). Two patients were excluded because they were unable to complete the task, and one patient was included because they were unable to pass the initial comprehension questions.
Non-participation	One participant dropped out of the fMRI study due to symptoms of claustrophobia in the scanner. Three lesion patients dropped out of the lesion patient study; one dropped out due to failing the initial comprehension test, and two dropped out early in the study due to fatigue.
Randomization	The experimental design of the fMRI study does not involve allocation of participants into different groups. Within the lesion study, data was analysed using two separate approaches. First, data from lesion patients was aggregated together (not allocated into separate groups) and analysed using voxel-wise regression alongside cluster correction methods (false discovery rate) to control for false positives. In the second analysis, lesion patients were allocated into two groups based on whether they were damaged within a region-of-interest pre-defined by the fMRI study. Potential group confounds such as task comprehension and age were controlled for during analyses.

Reporting for specific materials, systems and methods

We require information from authors about some types of materials, experimental systems and methods used in many studies. Here, indicate whether each material, system or method listed is relevant to your study. If you are not sure if a list item applies to your research, read the appropriate section before selecting a response.

Materials & experimental systems

n/a	Involved in the study
☒	☐ Antibodies
☒	☐ Eukaryotic cell lines
☒	☐ Palaeontology and archaeology
☒	☐ Animals and other organisms
☒	☐ Clinical data
☒	☐ Dual use research of concern
☒	☐ Plants

Methods

n/a	Involved in the study
☒	☐ ChIP-seq
☒	☐ Flow cytometry
☐	☒ MRI-based neuroimaging

Magnetic resonance imaging

Experimental design

Design type	Event-related design
Design specifications	The design consisted of 300 decision trials per subject split into 2 runs, with a 5 minute break between runs, and each run taking 25 minutes. Between trials, there was a jittered inter-trial interval of between 2.5 and 8 seconds. At the onset of the option offers, participants were required to wait 2 seconds before they could indicate their response by button press, to maximally dissociate decision and motor response events.
Behavioral performance measures	Button press choices (between three offers) were recorded, alongside response times. Performance was quantified as the number of points won during the session, and all participants performed well above chance.

Acquisition

Imaging type(s): Functional and structural

Field strength: 3T

Sequence & imaging parameters: Siemens scanner with a multiband accelerated echoplanar imaging sequence with the following parameters: voxel resolution 2.4 x 2.4 x 2.4 mm3, repetition time=1230 ms, echo time=30ms, flip angle=60 degrees, field of view=240mm, multiband acceleration factor=3, PAT factor=2, encoding direction=PA. A tilt angle of 30 degrees was used to minimize signal drop out in the orbitofrontal cortex (Deichmann et al., 2003).

Area of acquisition: Whole brain

Diffusion MRI: ☐ Used ☒ Not used

Preprocessing

Preprocessing software: Data were pre-processed using FMRIB's Software Library (FSL version 6.0.4), using the FEAT software tool (Woolrich et al. 2001). Gaussian spatial smoothing was applied with a full-width half-maximum of 5mm, and high pass temporal filtering was applied with a cut-off of 60s.

Normalization: Registration to standard space was performed using FLIRT (FMRIB's Linear Image Registration Tool) inside the FEAT software. Subject's T1-weighted structural image was used first to register the fMRI low resolution image. This was subsequently transformed to a standard T1-weighted image in MNI152 space.

Normalization template: Data were normalized to MNI152 space.

Noise and artifact removal: Functional data were motion corrected using rigid body registration to the central volume (Jenkinson et al., 2001, 2002). Cardiac and respiratory data were processed using FSL's Physiological Noise Modelling (PNM) tool to model the effects of physiological noise in the MRI data (Brooks et al. 2008).

Volume censoring: We detected and removed motion outliers using FEAT's fsl_motion_outliers tool.

Statistical modeling & inference

Model type and settings: Univariate analysis methods were used. A general linear model (GLM) was used to model BOLD activity in pre-whitened data space using parametric event-related regressors. Seven regressors of interest were included in the main GLM, predicting BOLD activity at the onset of the decision period. These regressors included participant choices, and model value for the different options.

Effect(s) tested: Standard higher-order statistical tests were performed on the group data estimates. GLM parameter estimates were first estimated at the level of run (first level), then combined within individuals as Fixed Effects (second level), and finally combined across subjects using FMRIB's Local Analysis of Mixed Effects (FLAME1+2; third level; Woolrich et al. 2004).

Specify type of analysis: ☐ Whole brain ☐ ROI-based ☒ Both

Anatomical location(s): Regions of interest in vmPFC, ACC, and striatum were selected on the basis of activity peaks from orthogonal regressors identified from the whole-brain analysis.

Statistic type for inference: Cluster-wise analyses were performed using a cluster probability threshold of p=0.05.

(See Eklund et al. 2016)

Correction: Multiple comparisons were corrected for using a Z statistic threshold of 3.1.

Models & analysis

n/a	Involved in the study
☐	☒ Functional and/or effective connectivity
☒	☐ Graph analysis
☒	☐ Multivariate modeling or predictive analysis

Functional and/or effective connectivity: We used standard general linear models (GLM) to investigate how neural activity varied parametrically with model regressors, performing statistics primarily on the group data estimates. We probed these effects further on an individual level by extracting region-of-interest activity and investigating the relationship between traits and fMRI activity on the individual level (Spearman's correlation).
